# Synergistic and distinct effects of expansive posture and nasal breathing on psychological and physiological self-regulation in adolescents

**DOI:** 10.1038/s41598-026-38917-6

**Published:** 2026-02-07

**Authors:** Xiaoying Yang, Zixi Liu, Zhenting Li, Haosheng Ye, Feiyan Luo, Yang Wu

**Affiliations:** 1 Department of Psychology, School of Education, Guangzhou University, 230 Wai Huan Xi Road, Guangzhou Higher Education Mega Center, Guangzhou, 510006 China; 2 Faculty of Education, Jinming Campus, Henan University, North Section of Jinming Avenue, Kaifeng, 450046 China; 3 Preschool Education College, Luoyang Normal University, No. 6, Jiqing Road, Luoyang, 471000 China; 4 Chen Deng Vocational and Technical School, B1 Lecong Road, Foshan, 528000 China

**Keywords:** Embodied self-regulation, Expansive posture, Breath regulation electrodermal activity, Autonomic nervous system, Adolescent mental health, Self-efficacy, Anxiety reduction, Health care, Neuroscience, Psychology, Psychology

## Abstract

Adolescents are vulnerable to anxiety and low self-efficacy due to heightened emotional reactivity and immature regulatory systems. Embodied interventions, such as posture adjustment and controlled breathing, have shown promise for enhancing psychological self-regulation, but their synergistic effects in adolescents remain unclear. This study examined the immediate and sustained effects of expansive posture, nasal breathing, and their combination on self-efficacy, anxiety, and autonomic nervous system (ANS) activity in adolescents aged 15–18 years. Participants completed the Trier Social Stress Test, received one of four interventions, and were assessed with self-report questionnaires (General Self-Efficacy Scale; State–Trait Anxiety Inventory-6; *n* = 138) and continuous electrodermal activity (EDA; *n* = 62) recorded during the intervention and a subsequent Stroop task. Results showed that expansive posture—alone or combined with nasal breathing—significantly increased self-efficacy, while all three active interventions reduced anxiety compared with controls. Physiological analyses revealed distinct patterns of ANS modulation: nasal breathing alone yielded more stable EDA profiles with lower variability—consistent with enhanced autonomic flexibility in HRV studies—while the combined intervention produced larger autonomic fluctuations, suggesting compensatory responses. These findings highlight the complementary benefits of posture and breathing strategies and support a phased “activate–then–stabilize” approach to adolescent self-regulation.

## Introduction

Adolescence, particularly the mid-to-late stage (ages 15–18), is a critical period marked by a heightened risk for mental health problems^1,2^. According to the World Health Organization approximately one in seven adolescents worldwide (around 14%) experience a mental health disorder^3^.This stage is characterized by increased emotional reactivity, alongside incomplete maturation of the neural circuits that support emotion and behavior regulation^4^.Due to the developmental lag of the prefrontal cortex relative to subcortical structures involved in emotional arousal, adolescents often struggle to regulate their emotions promptly and effectively in the face of stress or social evaluation, thereby increasing their vulnerability to anxiety and negative self-appraisal^2,5^. Persistent anxiety can further undermine self-efficacy in performance contexts^6^; for example, in test-anxious situations, increased effort may fail to fully offset the detrimental effects on performance^7^. Adolescents with low self-efficacy are more prone to feelings of helplessness and avoidance when confronted with academic or social challenges, which can perpetuate a maladaptive low-efficacy–high-anxiety emotional–cognitive cycle^8,9^. Therefore, identifying interventions that can both alleviate anxiety and enhance self-efficacy is of critical importance for supporting adolescent mental health and academic adaptation.

According to embodied cognition theory, bodily states are not only the outcome of mental activities but can also influence emotional and cognitive processing^10^. Within this framework, body posture and breathing patterns are regarded as important entry points for regulating psychological states through direct effects on the autonomic nervous system (ANS). Expansive postures—characterized by openness, an upright stance, and occupying more space—have been shown to enhance feelings of power, self-confidence, and willingness to take risks, effects that may be linked to sympathetic nervous system activation and endocrine changes associated with the stress response^11^. Moreover, Weineck found that adopting expansive gestures (e.g., raising both arms in a victory pose) significantly increased perceived power and reduced anxiety levels^12^, while Miragall demonstrated that women holding an expansive posture for just two minutes experienced a significant increase in positive affect^13^. These findings further support the potential role of expansive postures in emotional and stress regulation. At the same time, controlled breathing techniques—particularly nasal breathing exercises emphasizing deep ventilation and rhythmic regulation—have been found to effectively enhance vagal tone, promote parasympathetic dominance, and accelerate recovery from stress^14^. For instance, Chin and Kales reported that rhythmic nasal breathing significantly increased heart rate variability (HRV), an index reflecting enhanced parasympathetic activity, which was in turn associated with improvements in cognitive performance^15^. A substantial body of research has demonstrated that slow and regular breathing at approximately six breaths per minute (≈ 0.1 Hz) can significantly increase respiratory sinus arrhythmia (RSA) and the high-frequency component of heart rate variability (HF-HRV), reflecting enhanced cardiac vagal activity. These changes are often accompanied by improvements in baroreflex sensitivity, further promoting autonomic nervous system balance and facilitating recovery from stress^16,17^. At the physiological measurement level, electrodermal activity (EDA) is a well-established index for assessing autonomic nervous system (ANS) activity. Within this framework, skin conductance level (SCL) reflects relatively slow, tonic changes in overall arousal, whereas galvanic skin response (GSR) captures rapid, phasic fluctuations associated with specific events^18^. These measures are sensitive and real-time indicators of both immediate and sustained patterns of physiological regulation during and after an intervention, making them particularly valuable for elucidating the mechanisms underlying posture- and breathing-based interventions.

Although both posture and breathing regulation have shown promise in reducing anxiety, improving mood, and enhancing cognitive performance in adult populations, research in adolescents remains limited. Existing studies have primarily focused on single-mode interventions, with little mechanistic exploration of the synergistic effects between posture and breathing, and few have integrated psychological assessments with high-temporal-resolution physiological measures for comprehensive evaluation^18,19^. Moreover, adolescents’ emotional and physiological characteristics—such as heightened emotional reactivity and greater autonomic fluctuations^20^—may lead to immediate and delayed intervention effects that differ from those observed in adults, underscoring the need for targeted research. Against this backdrop, the present study aims to systematically examine the immediate and sustained effects of expansive posture and nasal breathing interventions (both individually and in combination) on adolescents’ anxiety levels, self-efficacy, and autonomic nervous system activity. By combining self-report questionnaires (General Self-Efficacy Scale, GSES^21^; State-Trait Anxiety Inventory, STAI-6^22^ with physiological measures such as electrodermal activity (EDA), this study seeks to elucidate the independent and interactive mechanisms of posture- and breathing-based interventions, thereby providing practical and scalable embodied strategies for promoting adolescent mental health.

To simultaneously examine both immediate and sustained (carry-over) regulatory effects of embodied interventions in an ecologically valid context, the present study employed a sequence of social-evaluative stress induction, brief embodied intervention, and secondary cognitive stressor. First, the Trier Social Stress Test (TSST) was used to reliably induce acute social-evaluative stress, elevating state anxiety, reducing self-efficacy, and increasing sympathetic arousal, thereby establishing a uniform dysregulated starting point for all participants. Immediately thereafter, a short (2–9.5 min) embodied intervention was delivered at peak distress to assess instantaneous psychological and physiological recovery. Finally, a computerized Stroop color-word interference task was administered as a secondary cognitive stressor to mimic ongoing academic pressure and to test whether regulatory benefits persist under renewed challenge, thereby allowing integrated evaluation of immediate recovery and sustained regulatory flexibility.

In summary, although both expansive posture and controlled nasal breathing have demonstrated regulatory effects in adults, their immediate and particularly sustained effects—and their interaction—in adolescents remain poorly understood. The present study therefore examined the psychological and physiological effects of expansive posture, nasal breathing, and their combination following acute social-evaluative stress. Based on embodied cognition and autonomic regulation theories, we tested the following preregistered hypotheses:

H1. Compared to a neutral control condition, all three embodied interventions (expansive posture alone, nasal breathing alone, and their combination) will significantly reduce state anxiety and increase general self-efficacy following the TSST.

H2. Nasal breathing (alone or combined with expansive posture) will confer additional and sustained benefits on autonomic flexibility, as evidenced by enhanced phasic electrodermal responsiveness during both the intervention phase and the subsequent Stroop cognitive stressor.

## Methods

### Participants

This study was preregistered on OSF (https://osf.io/tca2f/). The required sample size was estimated with G*Power (version 3.1.9.7) for a 4 (Group) × 2 (Time: pre- vs. post-test) mixed factorial design. Assuming a medium effect size (Cohen’s f = 0.25) and 80% power, the minimum total sample required was 48. Because multiple dependent variables were analyzed, we planned to control the family-wise Type I error with a Bonferroni correction and therefore increased the target sample size to 140. Accordingly, 150 participants were recruited by convenience sampling from two vocational high schools in Guangdong Province, China. After excluding 12 participants who did not meet the inclusion criteria, 138 adolescents (89 males, 49 females; age 15–18 years) remained for the initial questionnaire evaluation. Gender refers to biological sex as reported by the school registry.

To minimize confounding physiological factors, additional inclusion criteria were applied: (1) normal or corrected-to-normal vision; (2) right-handedness; (3) no history of psychiatric disorders, hypertension, or severe neurological conditions; (4) no current respiratory illness such as severe rhinitis, asthma, or common cold; (5) no intake of caffeine, tea, alcohol, or other substances affecting mental state within 24 h prior to the experiment; (6) adequate sleep the night before testing; and (7) female participants were tested outside their menstrual period. Following these stringent criteria, a final sample of 62 physiological participants qualified and completed the full experimental protocol. All final participants wore a wearable psychophysiological monitoring device (Psychorus wristband, HuiXin, Beijing, China) to record electrodermal activity (EDA). Written informed consent was obtained from legal guardians and written assent from all adolescent participants (15–18 years) prior to enrollment; the protocol was approved by the Ethics Committee of GuangZhou University and conducted in accordance with the Declaration of Helsinki and applicable regulations for research involving minors.

### Experimental procedure

Participants were randomly assigned into one of four experimental groups:

Group A (Expansive Posture, *n* = 13): Participants maintained an expansive posture (standing upright, chest expanded, hands on hips, legs apart to occupy more space) for 2 min.

Group B (Control, *n* = 13): Participants adopted a neutral sitting posture (sitting comfortably on a chair, feet shoulder-width apart, hands resting naturally on knees) for 2 min.

Group C (Nasal Breathing, *n* = 22): Participants sat comfortably, closed their eyes, and followed a guided nasal breathing exercise lasting approximately 9 min and 30 s. They were instructed not to control their inhalation but to prolong their exhalation as much as possible and exhale completely through the nasal cavity. Breathing frequency was monitored by the experimenter.

Group D (Expansive Posture & Nasal Breathing Combined, *n* = 14): Participants maintained the expansive posture described in Group A while simultaneously performing the nasal breathing exercise as in Group C. Participants were permitted brief adjustments if experiencing fatigue, after which they resumed the designated posture.

The study aimed to investigate the effects of expansive posture and nasal breathing interventions on adolescents’ self-efficacy and anxiety. Given that participants were adolescents aged 15–18, characterized by heightened sensitivity to social evaluation and immature emotional regulation capacities during mid-to-late adolescence, the Trier Social Stress Test (TSST) was employed to elicit emotional responses. The TSST involved preparing and delivering an impromptu public speech and completing a time-limited mental arithmetic task to induce performance-related anxiety^23^. Specifically, participants were given 5 min to prepare an impromptu speech on a topic of their choice, followed by delivering the speech face-to-face with two experimenters (one primary experimenter and one neutral observer, both maintaining impassive expressions). Immediately afterward, participants performed a mental arithmetic task, subtracting a two-digit number (less than 20) from a randomly generated four-digit number.

Immediately following the TSST, participants assessed their anxiety level using a 9-point scale (1 = extremely anxious; 9 = extremely calm). They then completed pre-intervention psychological measures, including the General Self-Efficacy Scale (GSES) for assessing perceived self-efficacy, and the short-form version of the State-Trait Anxiety Inventory (STAI-6) for evaluating anxiety levels.

**Fig. 1:**
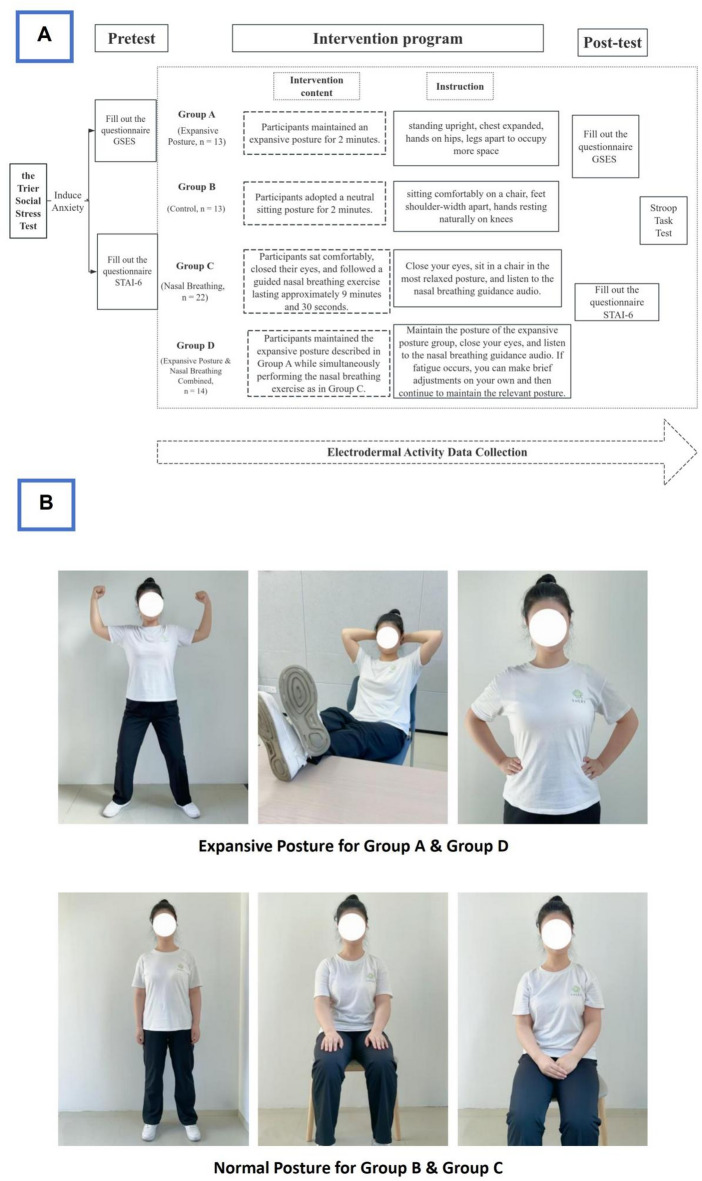
Experimental procedure.

After completing these pre-intervention questionnaires, participants received their assigned intervention according to their group. Immediately following the intervention, participants filled out the same psychological questionnaires (GSES and STAI-6) as post-intervention measures, and subsequently performed a computerized Stroop task^24^ (traditional Stroop paradigm involving responses to congruent and incongruent color-word stimuli). Throughout the experiment, participants wore a Psychorus wristband (HuiXin, Beijing, China), continuously recording their electrodermal activity (EDA) during the intervention and Stroop task phases. Precise timestamps were logged for subsequent analyses. A detailed experimental procedure is presented in Fig. 1, which depicts a posture demonstration performed by an adult volunteer and not an actual study participant. Informed consent for the publication of identifiable images was obtained from the volunteer. Written informed consent was obtained from this volunteer for publication of identifiable images in this open-access article.

### Physiological data processing

Electrodermal activity (EDA) data were preprocessed using the Ledalab software package^25,26^. First, the raw EDA signals were downsampled from 40 Hz to 10 Hz and smoothed using an 8-point Gaussian window to reduce noise artifacts. Subsequently, the EDA signals were decomposed into continuous tonic skin conductance level (SCL) and transient phasic skin conductance response (SCR) components using Continuous Decomposition Analysis (CDA). Specific parameters including mean values, peak amplitude, peak occurrence rates, and integrated SCR (iSCR) values were extracted separately for the intervention phase and the Stroop task phase. A total of 64 datasets for the intervention phase were initially obtained, requiring further quality control.

The detailed analysis procedures were as follows:

1. Peak detection:

Peak detection involved initially identifying peak onset (> 0.01µS) and offset (< 0µS) points from the phasic data. Corresponding peak intervals in the original unfiltered GSR data were subsequently matched to these detected onset-offset pairs, and the highest GSR amplitude between each pair was identified as the GSR peak value.

(2) Integrated SCR (iSCR) calculation:

To avoid subjectivity in threshold selection inherent to peak detection methods, the cumulative SCR effects were quantified independently through integration (iSCR). Specifically, cumulative SCR values were calculated within consecutive 10-second non-overlapping time windows, assuming a standardized sampling frequency of 1 Hz within each window.

(3) Extraction of GSR features during the intervention phase:

Fixed time windows based on precise timestamps were utilized to extract physiological response data. The difference between the maximum and minimum GSR values within each fixed window was computed to derive the peak amplitude. Higher peak amplitudes indicated greater physiological arousal levels during interventions. Additionally, the mean GSR and SCL values within the first two minutes of each intervention were calculated for visualization and analysis, enabling the comparison of physiological response trends across experimental groups.

(4) EDA data processing during stroop tasks:

Following peak detection, the peak occurrence rate (PKO) was determined by dividing the total number of peaks by the Stroop task duration, thus representing the frequency of physiological arousal fluctuations in response to the task stimuli. iSCR values during the Stroop task were used to reflect the overall transient physiological arousal, calculated as the integral of phasic drivers within the designated intervals. Given the typically skewed distribution of skin conductance responses, standardization of the iSCR data was performed using the following formula^25^:

$${\mathrm{iSCR}}\,=\,{\mathrm{log}}({\mathrm{1}}+\mid {\mathrm{ISCR}}\mid ) \times {\mathrm{sign}}\left( {{\text{ iSCR }}} \right)$$

(5) Statistical analysis

Data analyses were performed using SPSS 26.0 software. Two-way mixed factorial ANOVAs were conducted to test intervention effects on self-efficacy and anxiety level(using Type III sums of squares to appropriately handle the moderate imbalance in physiological subsample sizes). Simple-effects analyses and post hoc tests (LSD corrections) were applied where significant interactions were observed. For physiological data (GSR, SCL, SCR), one-way ANOVAs were conducted to compare group differences in mean EDA indices across intervention and Stroop-task phases, followed by LSD post-hoc multiple comparisons. Statistical significance was set at an alpha level of *p* < 0.05, with partial eta squared (*η*_*p*_²) used as a measure of effect size.

## Results

### Intervention effects on psychological measures

Two-way mixed ANOVAs (Group × Time) were conducted to examine the intervention effects on psychological outcomes, namely self-efficacy and anxiety^27^.

**Fig. 2:**
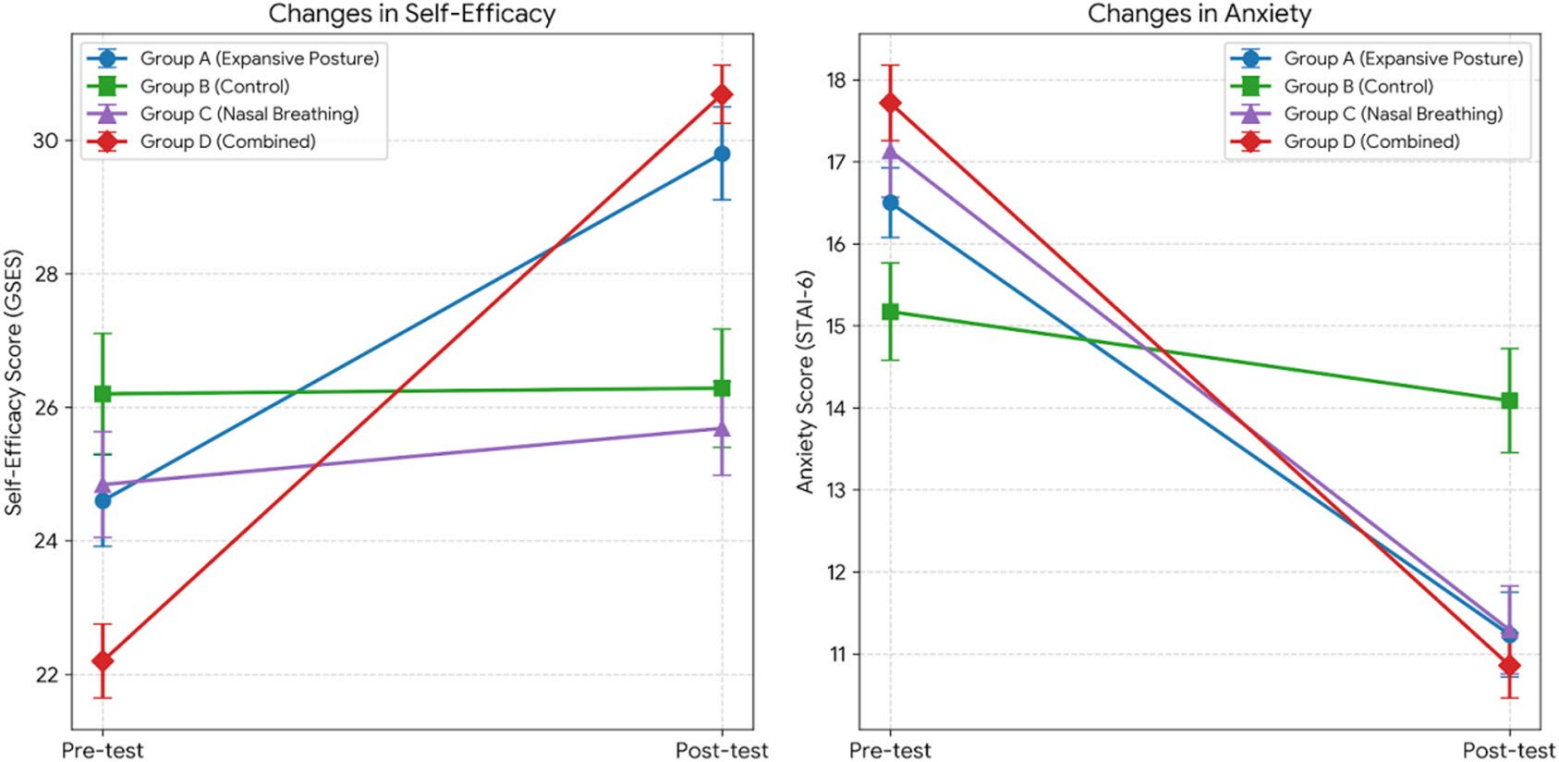
Changes in general self-efficacy and state anxiety pre- and post-intervention across experimental groups.

For self-efficacy (measured by GSES, questionnaire sample, *n* = 138), the main effect of Time was significant, indicating overall improvement in self-efficacy scores from pre- to post-intervention(Fig. 2), *F*(1,134) = 578.782, *p* < 0.001, *η*_*p*_^2^ = 0.812. Although the main effect of Group was not significant(*F*(1,134) = 1.251, *p* = 0.294), a significant interaction effect between Group and Time was observed, *F*(3,134) = 172.297, *p* < 0.001, *η*_*p*_^2^ = 0.794. Simple-effect analysis showed no significant differences among groups at pre-test (*p* > 0.050). However, at post-test, the Expansive Posture group and the Expansive posture & Nasal Breathing group showed significantly higher self-efficacy scores compared to Nasal breathing & Control group (*p* < 0.050).

For anxiety (measured by STAI-6, questionnaire sample, *n* = 138), the main effect of Time was significant, indicating a significant overall reduction in anxiety scores post-intervention(Fig. 2), *F*(3,134) = 570.511, *p* < 0.001, *η*_*p*_^2^ = 0.810. While the main effect of Group was not significant(*F*(3,134) = 0.384,*p* = 0.764), the interaction between Group and Time was significant, *F*(3,134) = 41.527, *p* < 0.001, *η*_*p*_^2^ = 0.482. Simple-effect analyses indicated no significant group differences at pre-test (*p* > 0.050). At post-test, the anxiety scores in the Control group were significantly higher than the other three intervention groups ( all *p* < 0.050).

### Intervention effects on physiological measures

**Fig. 3:**
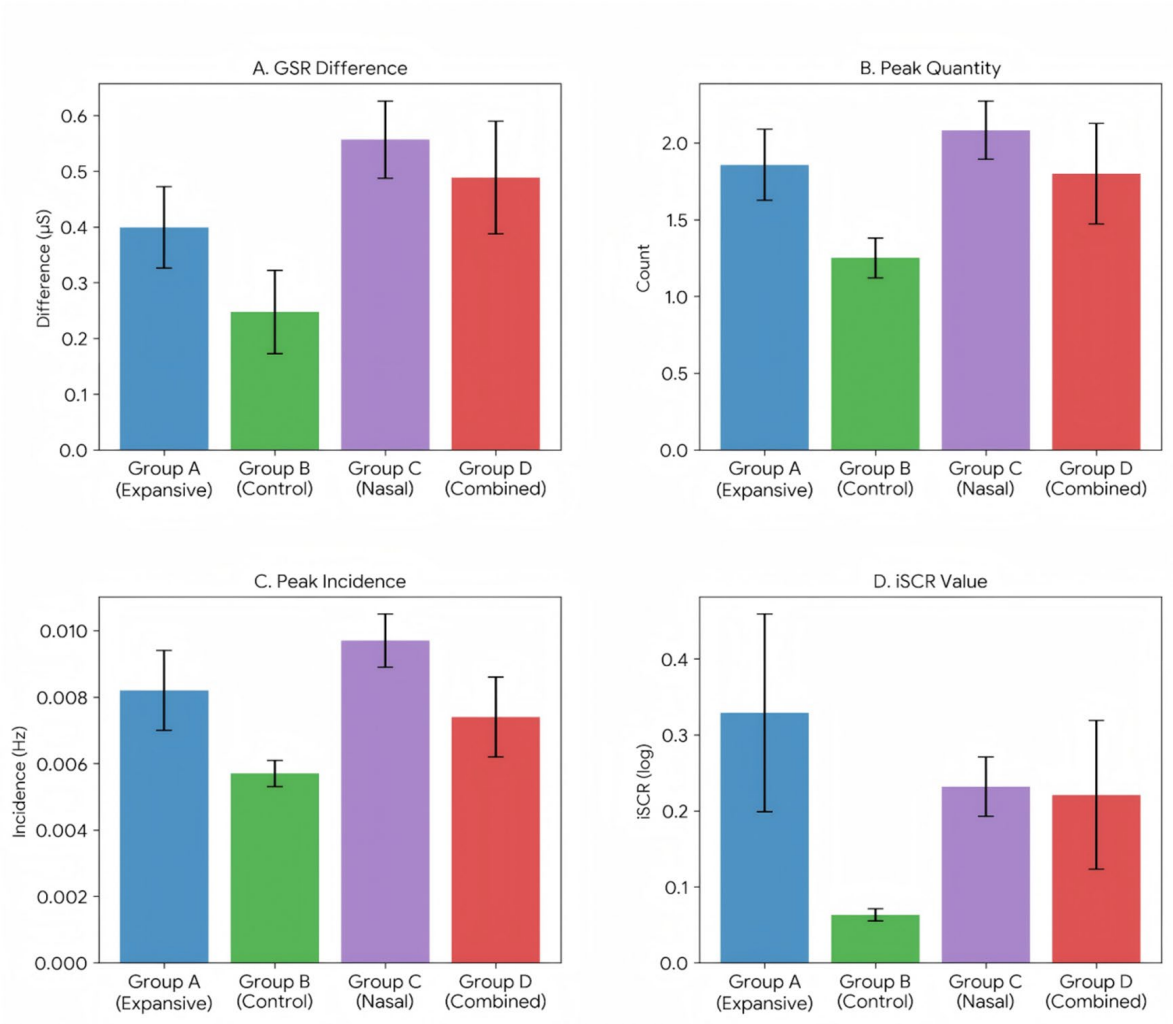
Comparison of Electrodermal Activity (EDA) indicators among different intervention groups (Mean ± SEM).

One-way ANOVAs were performed on electrodermal activity (EDA, physiological sample, *n* = 62) measures collected during both intervention and Stroop task phases. During interventions, significant group differences were found for mean GSR levels (*F*(3,58) = 5.910, *p* = 0.001, *η*_*p*_^2^ = 0.230) (Fig. 3). Post hoc LSD tests indicated that the Expansive Posture group showed significantly higher mean GSR levels compared to the Control group (*p* = 0.010), Nasal Breathing group (*p* = 0.017), and the Expansive posture & Nasal Breathing group (*p* < 0.001). Additionally, the Expansive posture & Nasal Breathing group exhibited significantly stronger physiological fluctuation (Fig. 3 GSR peak amplitude) compared to both the Expansive Posture group and Control group (*p* < 0.050). This suggests that this intervention modality elicited the strongest sympathetic nervous system activation. Moreover, regarding stress reactivity (ΔGSR, defined as the difference between the maximum and minimum GSR during the intervention phase), the Expansive posture & Nasal Breathing group exhibited significantly greater physiological fluctuations than both the Expansive Posture group (Δ = -0.31µS, *p* = 0.013) and the Control group (Δ= -0.55 µS, *p* < 0.001), suggesting the presence of a compensatory or rebound effect in autonomic nervous system regulation within this group.

During the Stroop tasks, a significant group effect was found for Peak Occurrence Rate (PKO) (*F*(3,58) = 6.532, *p* = 0.002, *η*_*p*_^2^ = 0.148) and iSCR values (*F*(3,58) = 7.459, *p* = 0.001,*η*_*p*_*²* = 0.088) (Fig. 3). Post hoc analyses revealed higher PKO and iSCR values for the Nasal Breathing group compared to the Control group (*p* < 0.050).

To determine the baseline level for each participant, the mean GSR value during the ten seconds prior to intervention onset was calculated. One-way ANOVA showed no significant differences in baseline GSR among the four groups. Therefore, GSR peak amplitude and SCL visualizations were used to evaluate the effects of the interventions.

**Fig. 4:**
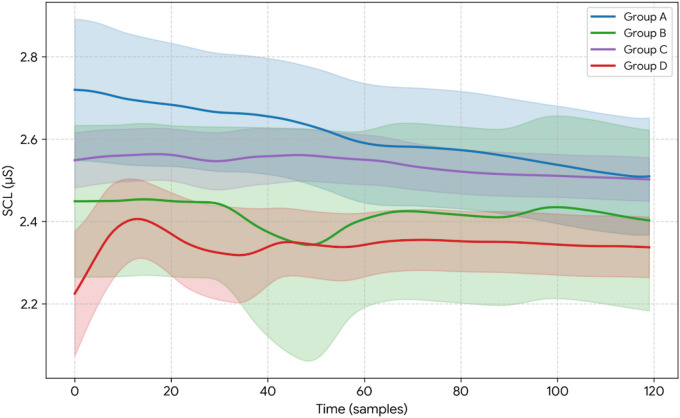
Visualization of mean SCL trends during the intervention phase.

Visualization of mean SCL trends during the intervention phase (see Fig. 4).

The Expansive Posture group (blue) exhibited the highest initial SCL (~ 2.73 µS), followed by a gradual decrease, indicating high initial sympathetic activation with subsequent adaptation and relaxation. The Control group (green) maintained a moderate and stable SCL (approximately 2.40–2.50 µS) throughout, with minimal fluctuation, suggesting the most stable physiological baseline among all groups. The Nasal Breathing group (purple) demonstrated slightly higher SCL than the Control group, with a steady and modest decrease, which may reflect ongoing autonomic regulation. The Expansive posture & Nasal Breathing group (red) showed the lowest initial SCL (~ 2.30 µS), followed by a transient increase and then a decline, indicating considerable baseline fluctuation and unstable physiological state at the onset of intervention.

**Fig. 5:**
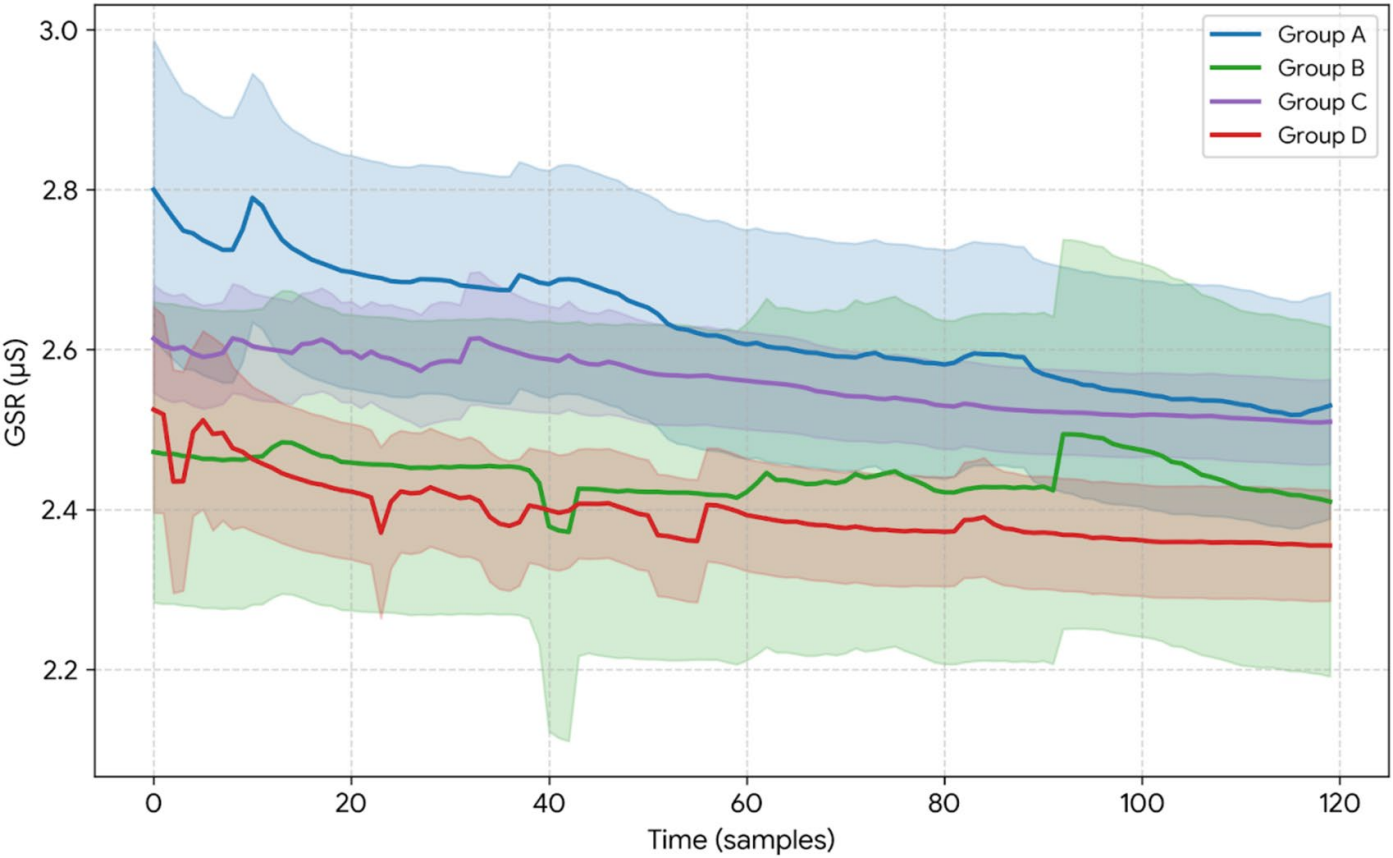
Visualization of mean GSR trends during the intervention phase.

Visualization of mean GSR trends during the intervention phase (see Fig. 5).

The Expansive Posture group (blue) showed the highest mean GSR (~ 2.80 µS), with a stepwise decline during the early intervention phase. A slight rebound (Δ + 0.06 µS) was observed at time point 11, possibly reflecting a transient external stimulus. By time point 111, the mean GSR decreased to approximately 2.50 µS, representing a reduction of about 10%, indicating a suggesting progressively reduced EDA arousal. The Control group (green) exhibited the largest amplitude of GSR fluctuations (peak-to-trough difference ~ 0.15 µS). An abnormal peak was detected at time point 81, which may be related to a temporary cognitive shift or emotional disturbance. The GSR ultimately stabilized at 2.40 µS, showing a 4% reduction from the initial value. The Nasal Breathing group (purple) maintained mean GSR values between 2.50 and 2.65 µS, with the smallest standard deviation (σ = 0.04). The iSCR values were significantly elevated, indicating a synergistic pattern of phasic autonomic activation with maintenance of tonic arousal—that is, greater phasic EDA responsiveness with low variability. This pattern is compatible with flexible autonomic regulation. The Expansive posture & Nasal Breathing group (red) displayed a “low baseline–high fluctuation” profile (ΔGSR = -0.55 µS, *p* < 0.001), which is characteristic of compensatory autonomic responses seen in chronic stress or high-stress populations. The significant increase in post-intervention GSR fluctuations may result from intervention parameters exceeding individual regulatory thresholds, thereby triggering a rebound effect in sympathetic nervous system activity.

In summary, our results demonstrate that different combinations of posture and breathing interventions elicit distinct autonomic modulation patterns. Expansive posture with normal breathing promotes efficient autonomic adaptation in individuals with high arousal. Notably, nasal breathing alone exhibited a more flexible EDA profile—low variability with robust phasic responses—and strong stress-buffering capacity, as reflected by stable, low-variability SCL/GSR and significantly elevated iSCR, facilitating a faster return to physiological homeostasis. By contrast, the combination of expansive posture and nasal breathing appeared to induce larger autonomic fluctuations and a potential rebound effect, evidenced by pronounced GSR variability and unstable SCL baselines; this pattern may reflect additive or threshold-crossing effects.

## Discussion

This study systematically investigated the immediate and sustained effects of expansive posture, nasal breathing, and their combination on psychological and physiological self-regulation in adolescents following social-evaluative stress. By integrating self-report measures with continuous electrodermal activity (EDA) monitoring, the findings provide robust support for the preregistered hypotheses, revealing distinct mechanisms through which body-based interventions modulate anxiety and self-efficacy.

Synergistic and Dissociable Psychological Benefits Consistent with Hypothesis 1 (H1), the results demonstrated that embodied interventions significantly improved psychological outcomes compared to the control condition. Specifically, all three intervention groups (expansive posture, nasal breathing, and their combination) effectively reduced state anxiety levels following the TSST. This aligns with accumulating evidence that both postural feedback^28^ and controlled breathing^29^ exert anxiolytic effects through bottom-up regulatory pathways.

However, the enhancement of self-efficacy was specific to interventions involving an expansive bodily component. Only the Expansive Posture and Combined (Posture & Nasal Breathing) groups showed significant increases in general self-efficacy. This supports the embodied cognition framework, suggesting that the physical act of occupying space (“power posing”) directly enhances perceived competence and agency^11,30^. Notably, the combined intervention produced the most pronounced improvements in both anxiety reduction and self-efficacy. This suggests a psychological synergy: posture primarily restores a sense of agency (countering helplessness), while nasal breathing targets physiological arousal (reducing anxiety), thereby creating an ideal “calm yet empowered” state for adolescents.

Nasal Breathing Promotes Sustained Autonomic Flexibility Physiological data collected during the subsequent cognitive stressor (Stroop task) supported Hypothesis 2 (H2). Groups incorporating nasal breathing (Nasal Breathing alone and the Combined group) exhibited significantly higher peak occurrence rates (PKO) and integrated skin conductance response (iSCR) values during the Stroop task compared to controls.

In the context of EDA, higher phasic responsiveness (reflected by iSCR/PKO) under cognitive load represents an adaptive capacity to rapidly mobilize physiological resources, rather than a state of chronic hyperarousal or blunted affect. This was evidenced by distinct patterns of autonomic modulation across groups. The Expansive Posture group showed the highest initial skin conductance level (SCL) and galvanic skin response (GSR) values, followed by a rapid decline, reflecting strong sympathetic activation and effective autonomic adaptation. In contrast, the Nasal Breathing group exhibited the most stable physiological profile—characterized by low variability and elevated iSCR—consistent with efficient stress buffering. Particularly noteworthy, the Combined group demonstrated a unique “low-baseline/high-fluctuation” autonomic pattern, potentially reflecting a compensatory or recalibration process. Furthermore, during the post-intervention Stroop task, groups receiving nasal breathing interventions showed higher PKO and iSCR values, highlighting the sustained regulatory advantage of nasal breathing in acute stress response and cognitive adaptation^31,32^. This confirms H2, indicating that nasal breathing practice not only facilitates immediate recovery but also prepares the autonomic nervous system for dynamic adaptation to subsequent challenges.

### Trajectories of self-efficacy and anxiety improvement

Using validated self-report questionnaires, this study systematically tracked changes in self-efficacy and anxiety across all intervention groups. The data revealed distinct trajectories of psychological improvement corresponding to each intervention modality.

Both the Expansive Posture and Combined groups exhibited significant increases in self-efficacy following the intervention. These findings align with embodied cognition theory, which posits that adopting open, confident postures enhances self-perceptions of competence and control^11,30^. The observed elevation in self-efficacy may reflect not only momentary boosts in confidence but also broader changes in perceived agency—a factor closely linked to resilience in adolescent populations^6,33^.

Regarding anxiety, all three intervention groups demonstrated significant reductions in self-reported anxiety levels compared to controls. The effect was most pronounced in the combined intervention group, highlighting the synergistic potential of integrated body-breath strategies. This is consistent with evidence supporting the anxiolytic effects of both posture and controlled breathing in reducing psychological stress and promoting emotional regulation.

Importantly, improvements in self-efficacy and anxiety paralleled the observed patterns in physiological indices, suggesting that embodied interventions simultaneously influence subjective experience and objective autonomic function These results underscore the value of multi-modal, context-sensitive self-regulation strategies for supporting adolescent mental health in real-world settings.

### Autonomic modulation in the initial intervention phase: group differences in SCL and GSR

Analysis of the first two minutes of physiological data offers critical insight into the immediate autonomic nervous system (ANS) responses elicited by different embodied interventions. Our findings revealed significant group differences in both skin conductance level (SCL) and galvanic skin response (GSR), reflecting distinct patterns of autonomic arousal indexed by EDA.

Specifically, the Expansive Posture group displayed the highest initial SCL and GSR, indicative of pronounced sympathetic activation at the onset of intervention. As the intervention progressed, this high arousal state rapidly declined, highlighting the strong adaptive capacity of this modality. Such a response pattern may be particularly suitable for situations requiring the rapid mobilization of resources, such as coping with acute stress. In contrast, the Nasal Breathing group showed the lowest variability in SCL/GSR alongside elevated iSCR, indicating a stable EDA profile with strong phasic responsiveness—a pattern compatible with improved autonomic regulation reported in breathing studies^14,34^. This physiological profile may support rapid recovery and emotional balance under stress.

Notably, the Combined group showed a distinct “low baseline–high fluctuation” pattern in ANS activity. Adolescents with high neuroticism or under chronic stress often exhibit heightened ANS responsivity and physiological variability^34,35^. For such individuals, interventions inducing greater physiological fluctuations might facilitate dynamic recalibration of the nervous system, potentially enhancing adaptive flexibility. However, this also increases the risk of excessive arousal or rebound effects, where physiological measures (e.g., GSR, SCL) may temporarily exceed baseline values post-intervention, potentially resulting in emotional instability^36,37^. Therefore, when working with adolescents showing distinct baseline physiological profiles or high reactivity, it is crucial to tailor intervention intensity and modality to minimize adverse autonomic reactions. Overall, the analysis of early physiological responses demonstrates that posture and breathing interventions modulate the ANS through distinct yet complementary mechanisms. Understanding these immediate effects is vital for optimizing the selection and sequencing of self-regulation strategies.

### Nasal breathing enhances sustained regulation: physiological evidence from response to subsequent cognitive stress

Beyond immediate intervention effects, this study examined whether the benefits persisted during subsequent cognitive challenges. Analysis of EDA during the Stroop task revealed distinct group differences in physiological adaptability under stress.

Groups receiving nasal breathing interventions—alone or in combination—showed higher PKO and iSCR values than the control condition. These results indicate stronger phasic EDA responsiveness and adaptability, reflecting a capacity for rapid physiological response and recovery during cognitive demands. This pattern is consistent with prior research suggesting that slow, controlled breathing is associated with improved autonomic flexibility and faster transitions between activation and recovery^14,16^. Notably, the control condition exhibited the lowest PKO, suggesting a less adaptive autonomic profile and potentially reduced flexibility in coping with acute cognitive stress. In contrast, the Nasal Breathing group displayed both stable baseline EDA (lower SCL/GSR variability) and robust dynamic phasic responses, suggesting that nasal breathing supports both baseline regulation and rapid reactivity—key features of healthy autonomic functioning.

These sustained effects underscore the value of integrating nasal breathing into stress management protocols for adolescents. By supporting both ongoing homeostasis and dynamic adaptability, nasal breathing interventions may facilitate not only acute recovery from stress but also optimal performance under pressure.

In practical applications, a phased approach to adolescent self-regulation is recommended: initially adopting a combined posture and breathing intervention to enhance self-efficacy and sympathetic activation for immediate stress coping, followed by a transition to nasal breathing alone to facilitate stabilization. For example, students experiencing significant anxiety before major examinations are advised to use the combined intervention prior to the test to rapidly boost confidence, then transition to nasal breathing alone to maintain self-regulation during the exam. Conversely, for routine self-regulation in contexts with lower daily stress or stable physiological baselines, students can directly use nasal breathing exercises.

### Practical implications for adolescent self-regulation interventions

The findings offer concrete guidance for the design and implementation of adolescent mental health interventions grounded in embodied self-regulation principles. Evidence supports the utility of both posture- and breathing-based interventions for enhancing self-efficacy, reducing anxiety, and optimizing autonomic flexibility. Importantly, these techniques are low-cost, non-invasive, and easily implemented in educational or community settings without specialized equipment.

The demonstrated value of integrating posture and nasal breathing strategies highlights the potential for multi-modal, stage-based interventions tailored to individual needs. For highly reactive adolescents, a phased approach—combining posture and breathing for acute activation followed by sustained nasal breathing for stabilization—may offer an optimal balance between activation and recovery. For the broader adolescent population, routine practice of nasal breathing can promote ongoing autonomic balance, emotional resilience, and cognitive performance.

Schools, mental health practitioners, and families are encouraged to incorporate brief posture and breathing exercises into daily routines, test preparation sessions, and stress management workshops. Baseline screening of physiological or psychological sensitivity may further enhance intervention personalization. Future school-based programs could leverage mobile or wearable technology to provide real-time feedback and adaptive training, supporting the scalable dissemination of embodied self-regulation practices. Collectively, these recommendations underscore the practical and scalable nature of posture and breathing interventions as accessible, evidence-based tools for promoting adolescent well-being.

### Limitations and future directions

Several limitations exist. First and foremost, intervention duration was confounded with type (2 min vs. 9.5 min), providing breathing groups 7.5 min of additional passive recovery after the TSST. This precludes fully separating specific intervention effects from natural recovery over time. Second, no physiological data were recorded during the TSST, preventing direct confirmation of comparable pre-intervention sympathetic activation. Third, the final physiological sample (*n* = 62) was substantially smaller than planned due to strict post-randomisation data-quality criteria, potentially reducing power for subtle autonomic effects despite large psychological effect sizes. Finally, the sample was male-dominated and drawn only from vocational schools, and long-term outcomes were not assessed. Future studies should equate intervention duration, record physiology throughout the TSST, use larger and more gender-balanced samples, and include longitudinal follow-up. Despite these limitations, the present findings demonstrate that brief embodied interventions—particularly nasal breathing—are highly promising for adolescent stress regulation. Given the still-maturing prefrontal-limbic circuitry characteristic of mid-to-late adolescence, simple, equipment-free techniques that directly modulate bodily states may be especially potent during this developmental window, offering rapid, scalable tools for real-world educational settings. Future research should employ duration-matched controls, measurement of nasal respiratory rate and depth of ventilation, full-session physiological monitoring, larger and more gender-balanced samples, and longitudinal designs to further establish efficacy and mechanisms.

## Conclusion

This study, integrating electrodermal activity and self-report questionnaires, systematically explored the mechanisms by which expansive posture, nasal breathing interventions, and their combination influence physiological and psychological outcomes as well as sustained effects in adolescents. Based on these findings, we propose a self-regulation protocol suitable for youth that effectively enhances self-efficacy, alleviates anxiety, and modulates autonomic nervous system function. The study underscores the complementary and synergistic benefits of combining posture and breathing strategies, as well as their practical application in individualized and context-sensitive interventions. Such embodied self-regulation approaches, integrating breath and posture, are practical, accessible, and well-suited for broad implementation in adolescent mental health promotion. Future research should further refine and disseminate these self-regulation strategies to enhance their adaptability and effectiveness across different contexts and populations.

## Data Availability

The informed consent obtained from all adolescent participants and their legal guardians prevents the public sharing of the research data. However, the datasets generated and/or analysed during the current study are available from the corresponding author ( [zixiliu560@gmail.com](https:/mailto: zixiliu560@gmail.com) ) upon reasonable request and with verification of ethical compliance.
